# Combined hydrophilic interaction liquid chromatography-scanning field asymmetric waveform ion mobility spectrometry-time-of-flight mass spectrometry for untargeted metabolomics

**DOI:** 10.1007/s00216-019-01790-6

**Published:** 2019-04-23

**Authors:** Katarzyna M. Szykuła, Joris Meurs, Matthew A. Turner, Colin S. Creaser, James C. Reynolds

**Affiliations:** 1 Centre for Analytical Science, Department of Chemistry, Loughborough University, Loughborough, LE11 3TU UK; 2 Advanced Materials and Healthcare Technology Division, School of Pharmacy, University of Nottingham, University Park, Nottingham, NG7 2RD UK

**Keywords:** Ion mobility, Differential mobility, Metabolomics, Bioanalytical methods, Separations, Mass spectrometry

## Abstract

Untargeted metabolite profiling of biological samples is a challenge for analytical science due to the high degree of complexity of biofluids. Isobaric species may also not be resolved using mass spectrometry alone. As a result of these factors, many potential biomarkers may not be detected or are masked by co-eluting interferences in conventional LC-MS metabolomic analyses. In this study, a comprehensive liquid chromatography-mass spectrometry workflow incorporating a fast-scanning miniaturised high-field asymmetric waveform ion mobility spectrometry separation (LC-FAIMS-MS) is applied to the untargeted metabolomic analysis of human urine. The time-of-flight mass spectrometer used in the study was scanned at a rate of 20 scans s^−1^ enabling a FAIMS CF spectrum to be acquired within a 1-s scan time, maintaining an adequate number of data points across each LC peak. The developed method is demonstrated to be able to resolve co-eluting isomeric species and shows good reproducibility (%RSD < 4.9%). The nested datasets obtained for fresh, aged, and QC urine samples were submitted for multivariate statistical analysis. Seventy unique biomarker ions showing a statistically significant difference between fresh and aged urine were identified with optimal transmission CF values obtained across the full CF spectrum. The potential of using FAIMS to select ions for in-source collision-induced dissociation is demonstrated for FAIMS-selected methylxanthine ions yielding characteristic fragment ion species indicative of the precursor.

**Electronic supplementary material:**

The online version of this article (10.1007/s00216-019-01790-6) contains supplementary material, which is available to authorized users.

**Figure:**
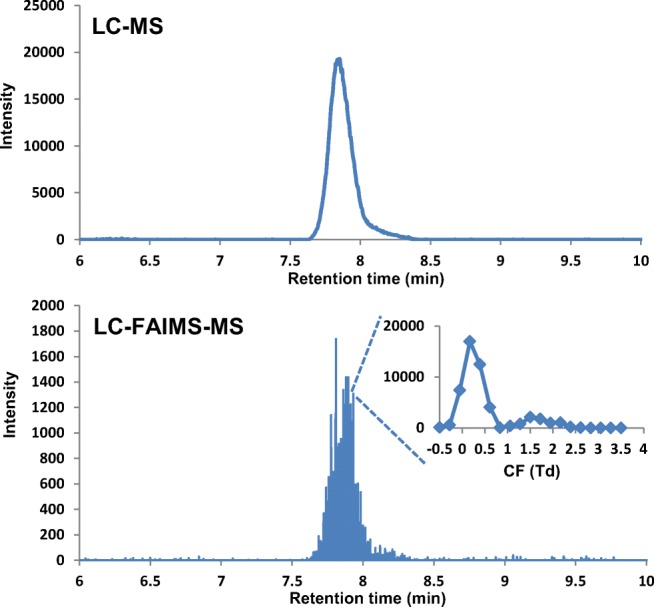
Graphical abstract

## Introduction

Metabolomic profiling remains a challenging task due the complexity of biological samples. The method most widely used in the metabolome composition analysis is liquid chromatography-mass spectrometry (LC-MS) [1–3]. Liquid chromatography allows the separation of analytes of interest according to their different partition coefficients, with long chromatographic run times required to separate ions with similar physicochemical properties [4]. Moreover, resolving isobaric species may not be achieved by mass spectrometry alone. These issues can be overcome by incorporating another separation technique such as ion mobility spectrometry (IMS) into the analysis. In this technique, ions in the gas phase are separated according to their mobility in an electric field [5]. The main advantage of IMS is its rapid time scale, so it is perfectly suited to be coupled to other separation and detection methods and there is growing interest in applying IMS to MS and LC-MS for investigating the composition of body fluids [6–8].

An alternative approach to conventional ion mobility spectrometry is field asymmetric waveform ion mobility spectrometry (FAIMS), also known as differential mobility spectrometry (DMS). Here, ion separation is based on their mobility in low and high electric fields, in contrast to conventional IMS, where only a low electric field is used [9–11]. In FAIMS [10], an alternating high- and low-voltage waveform, called the dispersion field (DF), is applied between two parallel electrodes. Ions travel between the plates in a saw-tooth trajectory and only those with a drift net zero variance will pass the electrodes. Ions which have positive or negative net variance will hit one of the electrodes and be neutralised. This effect can be counteracted by applying an additional field (compensation field (CF)), in the same direction as the DF. Any specific CF value allows only ions with a particular net drift to be transmitted through the electrodes. A wide range of CFs can be scanned in order to allow multiple ions to pass the drift cell and produce a differential mobility CF spectrum.

The hyphenation of FAIMS with MS and LC-MS is widely described in protein and peptide analysis, and has been reviewed by Swearingen et al. [12]. Its usage in small molecule studies is mainly focused on a targeted approach [13–21], and its application to untargeted metabolomics has received little attention. The first study that demonstrated metabolomic profiling of urine sample by FAIMS-MS was published by Beach et al. [22]. Their modification of a commercial FAIMS interface with cylindrical geometry electrodes resulted in obtaining good ion transmission and improved signal for the metabolites. The same group also investigated quantitation in the non-targeted analysis of urinary metabolites by FAIMS-MS [23]. The metabolic profiling of urine by combined LC-FAIMS-MS was first described in 2011 [24], also using a FAIMS device with cylindrical electrodes. The slow scan speed of the device did not allow the full CF range to be scanned within the timescale of an LC peak, so the FAIMS was stepped between six selected CFs, providing partial coverage of the metabolome. The hyphenation of hydrophilic interaction liquid chromatography (HILIC) with FAIMS-MS, using a miniaturised FAIMS device, was reported by Arthur et al. [25], where a detailed comparison of the HILIC-MS and HILIC-FAIMS-MS analysis of urine showed that FAIMS integration into the analysis significantly increased the number of molecular features detected using 11 CF steps. Recently, Wernish et al. [26] have published a study in which they analysed a mixture of more than 800 metabolites by 7 different chromatography columns. They compared the retention factors with CV values in order to estimate the degree of orthogonality between the retention time and compensation voltage, in both positive and negative ion modes. Higher orthogonality was obtained by using HILIC as a stationary phase than reverse phase (RP) chromatography.

In this work, we present a workflow for non-targeted metabolomic studies of urine samples by combining HILIC with fast-scanning miniaturised FAIMS and time-of-flight mass spectrometry (TOFMS). Nested LC-FAIMS-MS datasets were obtained by operating the TOFMS at 20 Hz, whilst the full compensation field range was scanned in 1 s, allowing several CF spectra to be acquired within the width of an LC peak. The chromatography run was performed in 9 min. The data obtained from fresh and aged urine samples were subjected to multivariate statistical analysis in order to detect differences between the two datasets.

## Materials and methods

### Sample collection

Four healthy participants (2 males, 2 females) were asked to collect their urine in ESS precleaned glass sample jars 250 mL (Cole-Parmer, London, UK) after night standing in the bladder. Urine samples were pooled together, mixed, and divided into two equal aliquots. One aliquot was further divided into 400 μL volumes in 2.0-mL micro-centrifuge tubes (Fisher Scientific, Loughborough, UK). These aliquots were stored at − 80 °C until analysed. The second aliquot was left open in a fume hood for 72 h at room temperature in order to age. Afterwards, the aged urine was aliquoted into 400 μL volumes and stored at − 80 °C. Quality control (QC) samples were prepared by mixing equal aliquots from fresh and aged urine samples.

### Chemicals and reagents

Acetonitrile and water LC-MS grade ammonium acetate HPLC grade were purchased from Fisher Scientific (Loughborough, UK). L-Carnitine was purchased from Sigma-Aldrich (St. Louis, USA), and hydrocortisone was purchased from Acros Organics (New Jersey, USA). ESI-L (low concentration tuning mix) and Biopolymer Reference Mass Kit HP-0321 were purchased from Agilent Technologies (Santa Clara, USA). A standard solution of L-carnitine and hydrocortisone (0.005 mg mL^−1^) was prepared in a mixture of acetonitrile:water (95:5, *v*/*v*).

### Sample preparation

Urine aliquots were thawed at room temperature for 20 min, then vortexed for 30 s, and sonicated for 1 min. In order to precipitate proteins, ice-cold acetonitrile was added to a urine aliquot (3:1 *v*/*v*). The mixture was then vortexed (30 s), sonicated (1 min), and left to stand for 10 min. Afterwards, the mixture was centrifuged at 12,200 rpm for 10 min. The supernatant was collected for further analysis and precipitated proteins were discarded. Six replicate samples of fresh and aged urine were prepared for LC-FAIMS-MS analysis.

### Instrumentation

**Fig. 1:**
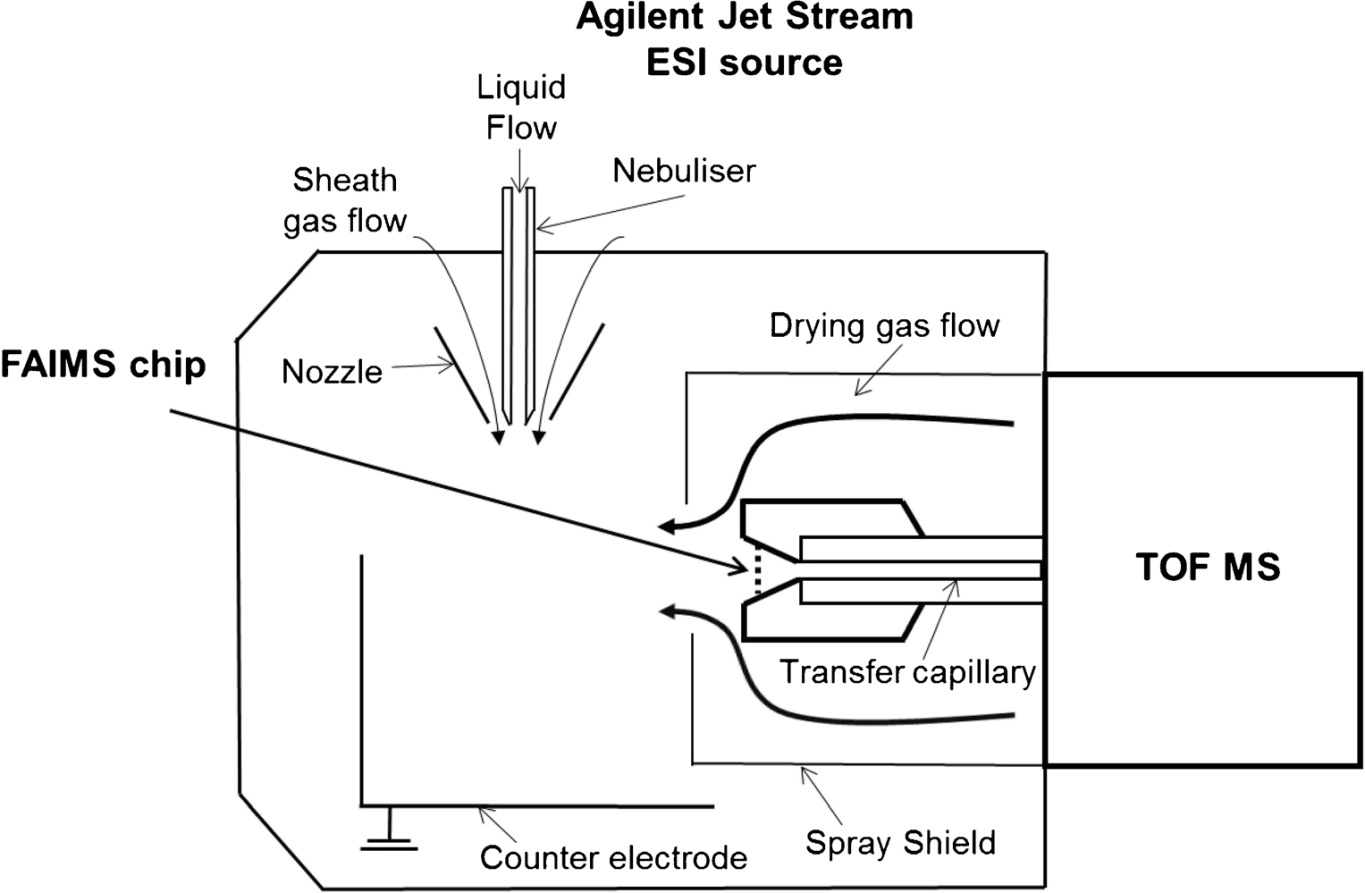
Schematic diagram of the FAIMS-MS interface [31]

LC-FAIMS-MS analyses were performed using an Agilent 1200 series liquid chromatograph interfaced to Agilent 6230 time-of-flight mass spectrometer (Agilent Technologies, Santa Clara, USA). The miniaturised chip-based FAIMS device (Owlstone Ltd., Cambridge, UK) was situated between Jet Stream electrospray source and mass spectrometer inlet (Fig. 1).

LC separation was performed on a 2.1 × 100 mm, 2.7 μm Poroshell 120 HILIC column (Agilent Technologies, Santa Clara, USA). For chromatographic separation, mobile phase A (10 mM ammonium acetate in water) and mobile phase B (10 mM ammonium acetate in acetonitrile:water (98:2 *v*/*v*)) were used. Urine extract (5 μL) was loaded onto the HILIC column and metabolites were eluted with a chromatographic gradient as follows: 97% B (0–0.2 min), decrease to 62% B (0.2–8.0 min), hold at 62% B for 1 min (8.0–9.0 min), increase to 97% B in 0.5 min (9.0–9.5 min), and hold for re-equilibration for 3.5 min. The total chromatographic cycle time was 13 min, at a flow rate at 0.5 mL min^−1^ and a column temperature of 35 °C.

Ion mobility separation was carried out at ambient pressure using a multichannel ultraFAIMS device with a 100-μm electrode gap, 78.1 mm trench length (span), and 700 μm ion path length [27]. The full CF range consisting of 19 steps from − 0.5 to 3.5 Td was acquired at rate 1 scan s^−1^, at a fixed DF of 240 Td, suitable for metabolomics studies [25]. The FAIMS device was controlled by the prototype software FAIMS Control (Agilent Technologies, Santa Clara, USA). The FAIMS scan was started automatically at 0.01 min into the chromatographic run. This operation was described in details by Arthur et al [25].

Mass spectrometry analysis was performed in positive ion mode, with a scan range from *m/z* 100 to 1500. The electrospray conditions were as follows: sheath gas flow 11 L min^−1^; sheath gas temperature 250 °C; drying gas flow 7 L min^−1^ and temperature 150 °C; nozzle and capillary voltage, 2000 V and 3500 V, respectively. The fragmentor voltage was set to 150 V (350 V for FISCID experiments) and the MS scan rate was 20 scans s^−1^.

### Data processing

The acquired nested LC-FAIMS-MS data were opened in Mass Hunter Qualitative Analysis software version B05.00 (Agilent Technologies, Santa Clara, CA, USA) and exported as MASCOT Generic Format (.mgf). These files were further converted to .mzXML format using MSConvert from ProteoWizard (v. 3.0.18178) [28]. The data were then loaded into Matlab R2017b (MathWorks, Inc., Natick, MA) and total ion current (TIC) chromatograms for each CF were extracted from the file, resulting in 20 TICs (19 CFs + re-initialisation step). Peak lists were obtained for each chromatogram with *m/z*, retention time (RT) and peak area determined for each feature. Mass values were binned to the second decimal place and the maximum allowed retention time deviation was fixed at 0.16 min. The data from quality control (*n* = 5), fresh (*n* = 6), and aged (*n* = 6) urine samples were subjected to principal component analysis (PCA) in order to detect the changes between datasets. Ions detected in less than 80% of all samples were excluded. Further, missing peak area values for included ions were replaced by nearest-neighbour imputation after which the data were zscore normalised. PCA scores and loadings plots were generated and Student’s *t* test was run to produce a list of ions with significant changes in peak areas between fresh and aged urine. The significance level was set at 0.05 and *p* values were corrected according to the Benjamini-Hochberg procedure [29]. All masses were internally recalibrated in Agilent Mass Hunter Software by using the L-carnitine standard.

## Results and discussion

Metabolic profiling of urine samples was performed by combining separation on a HILIC stationary phase with fast-scanning FAIMS (1 scan s^−1^) and a TOF mass spectrometer (20 scan s^−1^), resulting in the acquisition of three-dimensional (retention time, compensation field, and *m/z* datasets. Method development for the chromatographic separation was carried out using a solution of two organic compounds with different polarity [30]. The less polar compound, hydrocortisone, was eluted at the very beginning of the chromatography run (0.802 min) while the quaternary ammonium compound L-carnitine was strongly retained on HILIC column and eluted with a retention time of 8.21 min, towards the end of the 9-min chromatographic run.

The pooled urine extract was analysed by LC-FAIMS-MS at a range of injection volumes (1–20 μL) in triplicate and peak areas for selected ions were plotted against their corresponding injection volumes (see Electronic Supplementary Material (ESM) Fig. S1), showing *R*^2^ > 0.98. The reproducibility of the developed method was verified by calculating the relative standard deviation (RSD %) for peak areas and retention times (Table 1). RSD% were below 5% for peak areas and below 0.2% for retention time, which is suitable for metabolomic applications, allowing the acquisition of nested LC-FAMS-MS data without increasing the LC-MS run time.

**Table 1:** Reproducibility of peak areas and retention times for selected metabolite ions (5 μL injection volume, *n* = 3)

*m/z* ± 0.02	Peak area	Peak area RSD %	RT [min]	RT RSD%	CF [Td]
114.066	45,762	1.5	3.43	0.13	1.30
121.072	147,773	3.8	1.57	0.04	− 0.05
265.119	6441	4.9	4.12	0.05	2.61
302.232	4318	4.0	5.12	0.13	2.61
679.014	9438	4.2	6.14	0.14	2.39

**Fig. 2:**
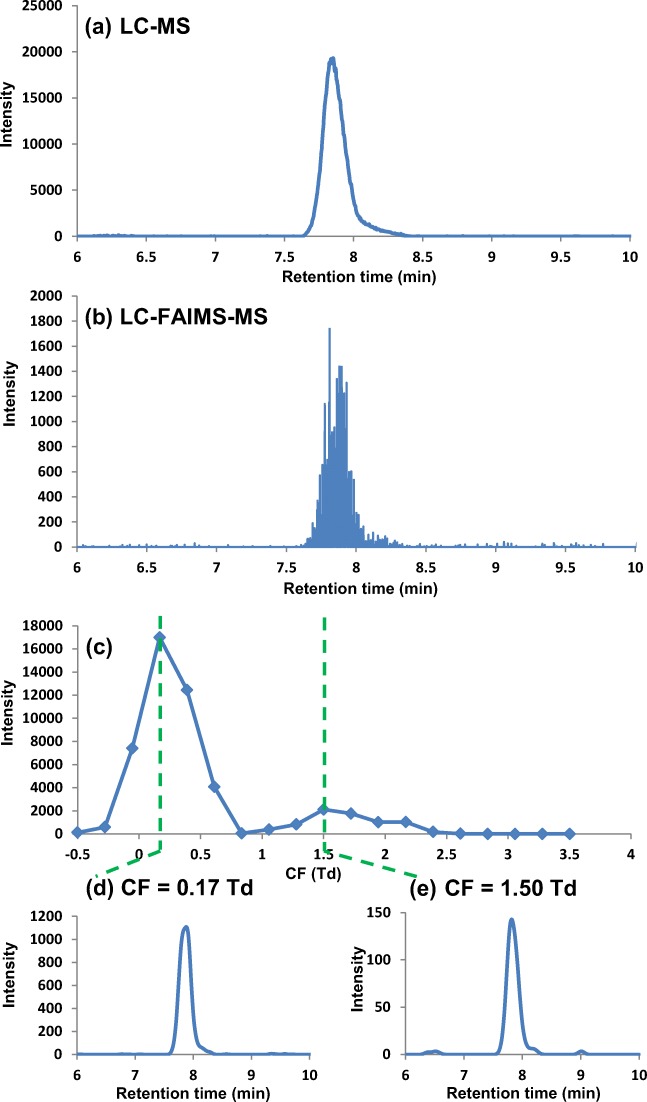
(**a**) LC-MS extracted ion chromatogram (*m/z* 137.071) for urine, (**b**) LC-FAIMS-MS extracted ion chromatogram (*m/z* 137.071) for the same urine sample over the full CF range, (**c**) CF spectrum (CF − 0.5 to 3.5 Td) and (**d**) extracted ion chromatograms (*m/z* 137.071) at CF 0.17 Td (lower left) and at (**e**) CF 1.5 Td (lower right)

The incorporation of FAIMS into the HILIC-MS analysis (HILIC-FAIMS-MS) has been shown to result in increased peak capacity [25]. This includes the resolution of ions with the same exact mass and retention time, demonstrated in Fig. 2, which shows the extracted ion chromatogram for *m/z* 137.071 (C_7_H_9_N_2_O; tentative annotation methylnicotinamide) [32]. Using LC-MS analysis, a single peak was detected (Fig. 2(a)) at a retention time of 7.85 min. However, the addition of FAIMS to the LC-MS analysis (Fig. 2(b)) results in a FAIMS spectrum for *m/z* 137.071, which presents two peaks, one at CF 0.17 Td (RT 7.88 min) and the second peak at CF 1.5 Td (RT 7.80 min) (Fig. 2(c, d)). This suggests that two species, possibly isomers, with the same retention time and *m/z* were detected by FAIMS separation.

The entire LC-FAIMS-MS workflow for an untargeted metabolomic study was evaluated by analysing six replicate samples of fresh and aged urine in order to detect the differences between the two datasets. A quality control (QC) sample, prepared by mixing fresh and aged urine, was used for column conditioning (*n* = 5) before the analysis and then during the analysis after every 3 samples. The acquired data were converted into easy readable .mzXML format and further processed in Matlab. The data from each file were separated into 20 individual chromatograms, one each for the 19 steps in the CF scan plus one for the instrument re-initialisation, from which the peak lists were obtained. Subsequently, the data from QC samples, fresh and aged urine were subjected to multivariate statistical analysis.

**Fig. 3:**
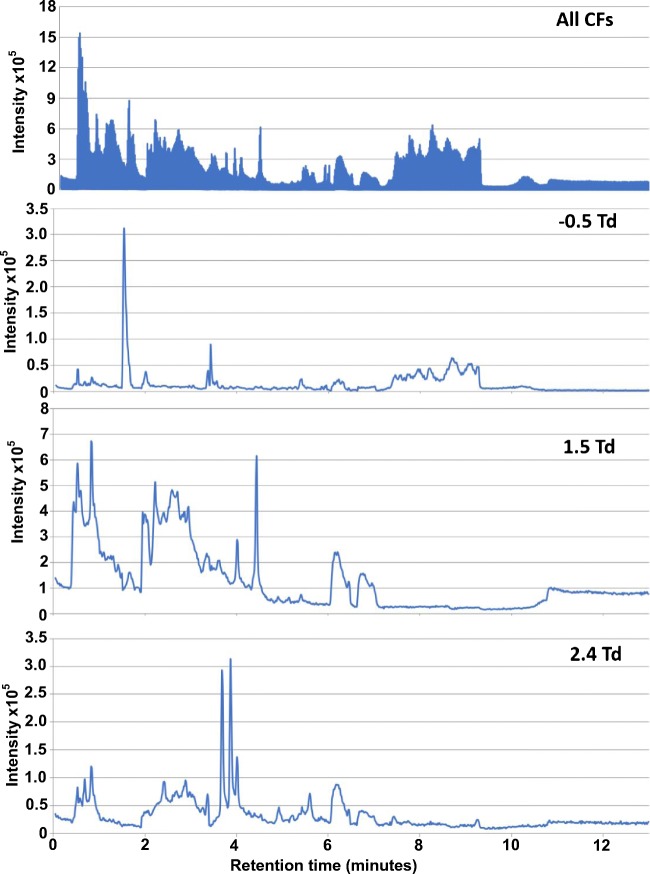
LC-FAIMS-MS total ion chromatograms for a urine extract at selected CFs

**Fig. 4:**
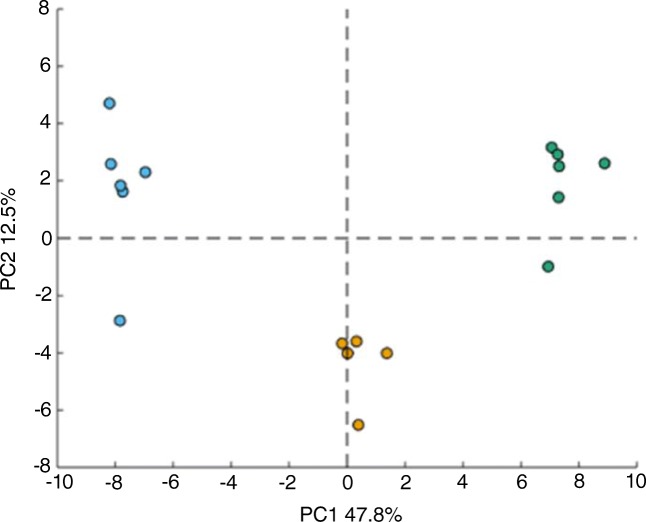
PCA plots of urine samples analysed by HILIC-FAIMS-MS at a CF of 0.39 Td, containing fresh (blue), aged (green), and quality control samples (orange)

**Fig. 5:**
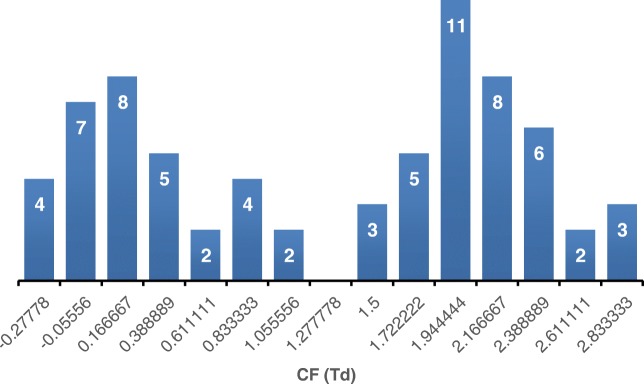
The number of ions with significant change in abundance between fresh and aged urine samples detected at each CF step from − 0.28 to 2.83 Td

The effect of different compensation fields on the TIC profile of urine sample is presented in Fig. 3. It can be seen that selecting individual CFs results in different chromatographic profiles derived from the urine sample. PCA was performed for fresh, aged, and QC samples and the PC1/PC2 plots obtained from the nested FAIMS-MS dataset obtained at a CF of 0.39 Td are presented in Fig. 4 showing a clear separation of the 3 sample groups. Separation of fresh and aged urine samples can be observed on most of the plots, with QC samples clustering in the middle (ESM Fig. S2). The nature of the PCA plots changes with the compensation field, which corresponds to the diverse information obtained at every CF step. Figure 5 shows the number of unique ions with significant change in abundance between fresh and aged urine samples detected at particular CF values. The number of ions detected varies between CFs, but the highest numbers of up- and down-expressed metabolite ions are observed at CF ~ 0.2 and ~ 2.0 Td (Fig. 5).

**Fig. 6:**
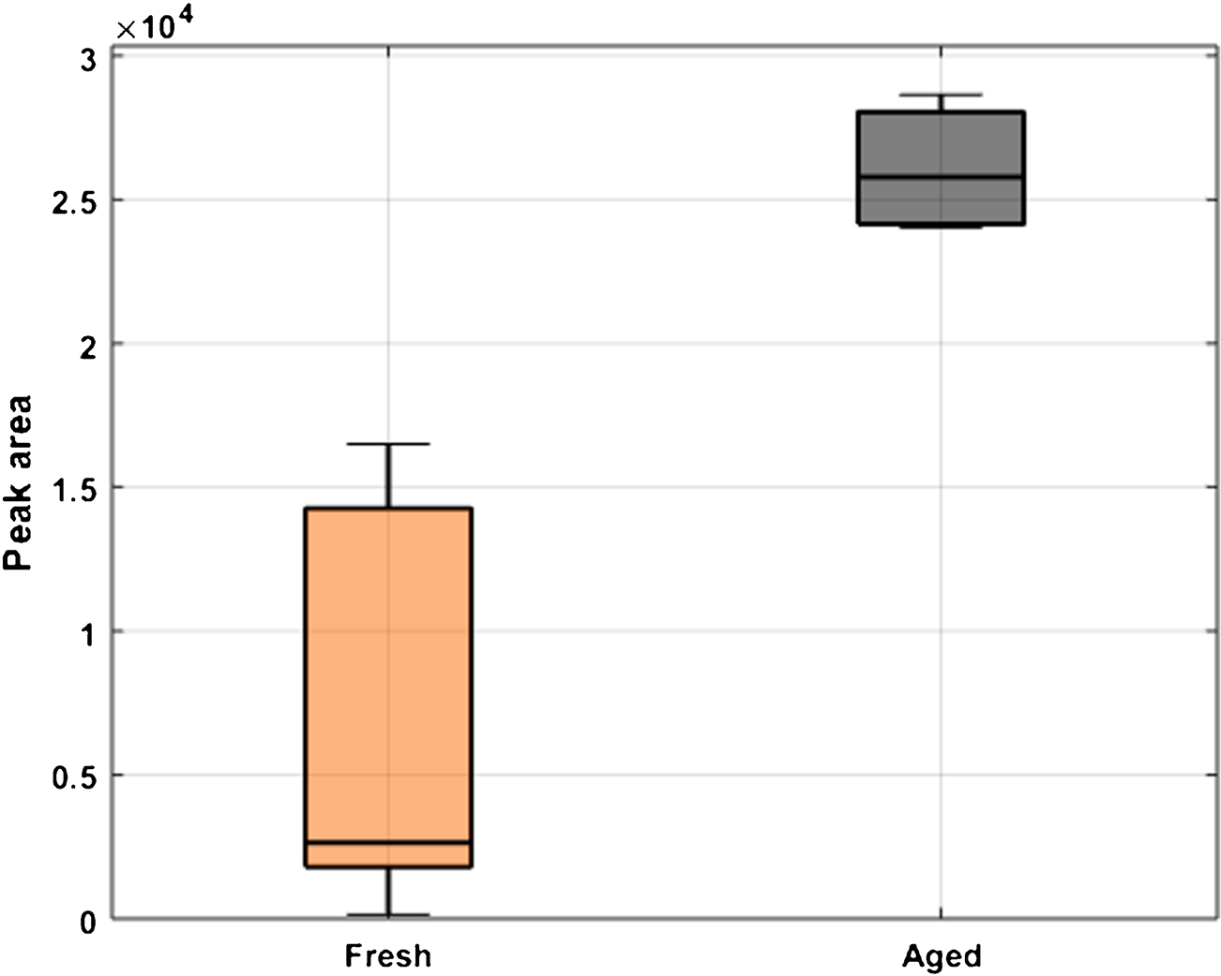
Box plot of ion 298.1145, showing the differences of peak areas for fresh and aged urine samples; RT 2.6 min, CF 1.94 Td

In total, there were 70 ions showing significant changes in abundance between fresh and aged urine with *p* value < 0.05. The majority of ions increased in concentration with the age of the samples (55), whilst only 15 features showed a significant decrease (ESM Fig. S3). ESM Table S1 shows the entire list of 70 metabolites which showed significant changes with their *m/z*, retention times, median fold change, *p* values, and the CF at which they were detected. It should be noted that there is no correlation between the masses, retention time, and CF values which clearly shows that these three analytical techniques are highly orthogonal to each other. The largest median fold change was obtained for *m/z* 298.1145 (C_11_H_16_N_5_O_5_; tentative annotation *N*-methylguanosine) [33] (Fig. 6). Its increase may be explained by the fact that *N*-methylguanosine is a degradation product of tRNA [33] and can increase as the biofluid is ageing.

**Fig. 7:**
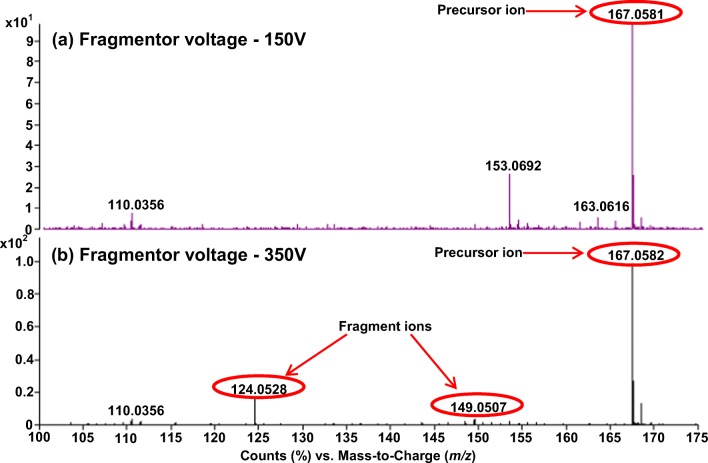
FAIMS selected (CF = 1.94 Td) in-source collision-induced dissociation mass spectra (FISCID-MS) acquired at fragmentor voltages **a** 150 V and **b** 350 V

The hyphenation of FAIMS with in-source collision-induced dissociation (CID) in the TOF mass spectrometer inlet enables FAIMS-selected ions which pass through the FAIMS electrodes to be fragmented prior to mass analysis. This method, described as FISCID-MS, simplifies the fragment ion mass spectrum which results in better ion identification [32]. Figure 7 shows two FAIMS selected (CF = 1.94 Td) mass spectra from a LC peak eluting at 1.25 min for the *m/z* range 100–180, acquired at two fragmentor voltages: 150 and 350 V. It can be seen that by applying the higher fragmentor voltage, two fragment ions (*m/z* 149.050 and 124.052) are derived from the precursor ion with *m/z* 167.058. This result can be compared with the Human Metabolome Database [33], in the similar way as it is done using tandem mass spectrometry analysis (MS/MS), which facilitates the ion identification as methylxanthine.

## Conclusions

Fast-scanning FAIMS was combined with HILIC-MS allowing nested LC-FAIMS-MS datasets to be acquired for the untargeted metabolomic study of urine. The incorporation of FAIMS into the LC-MS analysis resulted in increased peak capacity due to the high degree of orthogonality of FAIMS, HILIC, and MS separation. The developed LC-FAIMS-MS workflow was evaluated for fresh and aged urine samples. Multivariate statistical analysis of urine datasets demonstrated the clear separation of fresh urine and urine after 72 h, at several CF values. As various CFs allowed different metabolites to be transmitted, the detected metabolites will differ across the CF spectrum. The total number of ions whose concentration has changed significantly includes 55 up- and 15 downregulated metabolite species. The highest numbers of ions were detected at CFs ~ 0.2 and ~ 2.0 Td, with *m/z* 298.1145 (tentative annotation *N*-methylguanosine) showing the largest median fold change between samples. Moreover, the FISCID-MS method enables the generation of fragment ions which can aid in metabolite identification. To summarise, the developed workflow of urine sample analysis by LC-FAIMS-MS has shown that this method has a potential for untargeted metabolomic studies and biomarker discovery. We have demonstrated one complete workflow that uses HILIC chromatography, but the approach could be extended to FAIMS-MS combined with other chromatography and separation techniques used in metabolomics. Furthermore, it can be applied to other biological matrices as well as other omics applications.

## Electronic supplementary material

ESM 1 (PDF 620 kb)

## References

[1] Yin P, Xu G (2014). Current state-of-the-art of nontargeted metabolomics based on liquid chromatography-mass spectrometry with special emphasis in clinical applications. J Chromatogr A.

[2] Vorkas PA, Isaac G, Anwar MA, Davies AH, Want EJ, Nicholson JK (2015). Untargeted UPLC-MS profiling pipeline to expand tissue metabolome coverage: application to cardiovascular disease. Anal Chem.

[3] Gagnebin Y, Tonoli D, Lescuyer P, Ponte B, de Seigneux S, Martin PY (2017). Metabolomic analysis of urine samples by UHPLC-QTOF-MS: impact of normalization strategies. Anal Chim Acta.

[4] Schrimpe-Rutledge AC, Codreanu SG, Sherrod SD, McLean JA (2016). Untargeted metabolomics strategies—challenges and emerging directions. J Am Soc Mass Spectrom.

[5] Eiceman GA, Karpas Z, Hill HH (2016). Ion mobility spectrometry.

[6] Dwivedi P, Schultz AJ, Hill HH (2010). Metabolic profiling of human blood by high-resolution ion mobility mass spectrometry (IM-MS). Int J Mass Spectrom.

[7] Harry EL, Weston DJ, Bristow AWT, Wilson IA, Creaser CS (2008). An approach to enhancing coverage of the urinary metabonome using liquid chromatography-ion mobility-mass spectrometry. J Chromatogr B.

[8] Malkar A, Devenport NA, Martin HJ, Patel P, Turner MA, Watson P (2013). Metabolic profiling of human saliva before and after induced physiological stress by ultra-high performance liquid chromatography–ion mobility–mass spectrometry. Metabolomics..

[9] Buryakov IA, Krylov EV, Nazarov EG, Rasulev UK (1993). A new method of separation of multi-atomic ions by mobility at atmospheric pressure using a high-frequency amplitude-asymmetric strong electric field. Int J Mass Spectrom Ion Process.

[10] Shvartsburg AA (2008). Differential ion mobility spectrometry: nonlinear ion transport and fundamentals of FAIMS.

[11] Purves RW, Guevremont R (1999). Electrospray ionization high-field asymmetric waveform ion mobility spectrometry-mass spectrometry. Anal Chem.

[12] Swearingen KE, Moritz RL (2012). High-field asymmetric waveform ion mobility spectrometry for mass spectrometry-based proteomics. Expert Rev Proteomics.

[13] McCooeye MA, Ells B, Barnett DA, Purves RW, Guevremont R (2001). Quantitation of morphine and codeine in human urine using high-field asymmetric waveform ion mobility spectrometry (FAIMS) with mass spectrometric detection. J Anal Toxicol.

[14] Varesio E, Le Blanc JCY, Hopfgartner G (2012). Real-time 2D separation by LC × differential ion mobility hyphenated to mass spectrometry. Anal Bioanal Chem.

[15] Smith RW, Toutoungi DE, Reynolds JC, Bristow AWT, Ray A, Sage A (2013). Enhanced performance in the determination of ibuprofen 1-β-O-acyl glucuronide in urine by combining high field asymmetric waveform ion mobility spectrometry with liquid chromatography-time-of-flight mass spectrometry. J Chromatogr A.

[16] Chen PS, Chen SH, Chen JH, Haung WY, Liu HT, Kong PH (2016). Modifier-assisted differential mobility–tandem mass spectrometry method for detection and quantification of amphetamine-type stimulants in urine. Anal Chim Acta.

[17] Arthur KL, Turner MA, Brailsford AD, Kicman AT, Cowan DA, Reynolds JC (2017). Rapid analysis of anabolic steroid metabolites in urine by combining field asymmetric waveform ion mobility spectrometry with liquid chromatography and mass spectrometry. Anal Chem.

[18] Chen Z, Coy SL, Pannkuk EL, Laiakis EC, Hall AB, Fornace AJ (2016). Rapid and high-throughput detection and quantitation of radiation biomarkers in human and nonhuman primates by differential mobility spectrometry-mass spectrometry. J Am Soc Mass Spectrom.

[19] Chen Z, Coy SL, Pannkuk EL, Laiakis EC, Fornace AJ, Vuoros P (2018). Differential mobility spectrometry-mass spectrometry (DMS-MS) in radiation biodosimetry: rapid and high-throughput quantitation of multiple radiation biomarkers in nonhuman primate urine. J Am Soc Mass Spectrom.

[20] Arthur KL, Wilson LS, Turner MA, Lindley MR, Reynolds JC, Creaser CS. The determination of salivary oxipurines before and after exercise by combined liquid chromatography-field asymmetric waveform ion mobility spectrometry-time-of-flight mass spectrometry. Int J Ion Mobil Spectrom. 2018. 10.1007/s12127-018-0232-4.

[21] Porta T, Varesio E, Hopfgartner G (2013). Gas-phase separation of drugs and metabolites using modifier-assisted differential ion mobility spectrometry hyphenated to liquid extraction surface analysis and mass spectrometry. Anal Chem.

[22] Beach DG, Gabryelski W (2011). Nontarget analysis of urine by electrospray ionization-high field asymmetric waveform ion mobility-tandem mass spectrometry. Anal Chem.

[23] Beach DG, Gabryelski W (2013). Linear and nonlinear regimes of electrospray signal response in analysis of urine by ESI-FAIMS-MS and implications for nontarget quantification. Anal Chem.

[24] Harry E. The development of ion mobility-mass spectrometry for complex mixture analysis. Loughborough University PhD thesis. 2011. https://dspace.lboro.ac.uk/2134/9119. Accessed 6 Sept 2018.

[25] Arthur KL, Turner MA, Reynolds JC, Creaser CS (2017). Increasing peak capacity in non-targeted omics applications by combining full scan field asymmetric waveform ion mobility spectrometry with liquid chromatography-mass spectrometry. Anal Chem.

[26] Wernisch S, Afshinnia F, Rajendiran T, Pennathur S (2018). Probing the application range and selectivity of a differential mobility spectrometry–mass spectrometry platform for metabolomics. Anal Bioanal Chem.

[27] Gabelica V, Shvartsburg AA, Afonso C, Barran P, Benesch JLP, et al. Recommendations for reporting ion mobility mass spectrometry measurements. Mass Spectrom Rev. 2019. 10.1002/mas.21585. 10.1002/mas.21585 PMC6618043 30707468

[28] Kessner D, Chambers M, Burke R, Agus D, Mallick P (2008). ProteoWizard: open source software for rapid proteomics tools development. Bioinformatics.

[29] Hochberg YB, Benjamini Y (1995). Controlling the false discovery rate: a practical and powerful approach to multiple testing. J R Stat Soc Ser B.

[30] Malkar A, Wilson E, Harrrison T, Shaw D, Creaser CS (2016). Untargeted metabolic profiling of saliva by liquid chromatography-mass spectrometry for the identification of potential diagnostic biomarkers of asthma. Anal Methods.

[31] Brown LJ, Smith RW, Toutoungi DE, Reynolds JC, Bristow AWT, Ray A (2012). Enhanced analyte detection using in-source fragmentation of field asymmetric waveform ion mobility spectrometry-selected ions in combination with time-of-flight mass spectrometry. Anal Chem.

[32] Roux A, Xu Y, Heilier JF, Olivier M-F, Ezan E, Tabet J-C (2012). Annotation of the human adult urinary metabolome and metabolite identification using ultra high performance liquid chromatography coupled to a linear quadrupole ion trap-orbitrap mass spectrometer. Anal Chem.

[33] Human Metabolome Database. http://www.hmdb.ca/. Accessed 24 Jul 2017.

