# Supplementary material for: Combined hydrophilic interaction liquid chromatography-scanning field asymmetric waveform ion mobility spectrometry-time-of-flight mass spectrometry for untargeted metabolomics
Source: Anal Bioanal Chem. 2019 Apr 23;411(24):6309–17. doi: 10.1007/s00216-019-01790-6 (PMC6718375; doi:10.1007/s00216-019-01790-6)
Supplement: Supplementary file 1 — (PDF 620 kb) [file 216_2019_1790_MOESM1_ESM.pdf]

Analytical and Bioanalytical Chemistry

Electronic Supplementary Material

Combined hydrophilic interaction liquid chromatography-scanning field asymmetric waveform ion mobility spectrometry-time-of-flight mass spectrometry for untargeted metabolomics

Katarzyna M. Szykuła, Joris Meurs, Matthew A. Turner, Colin S. Creaser,
James C. Reynolds

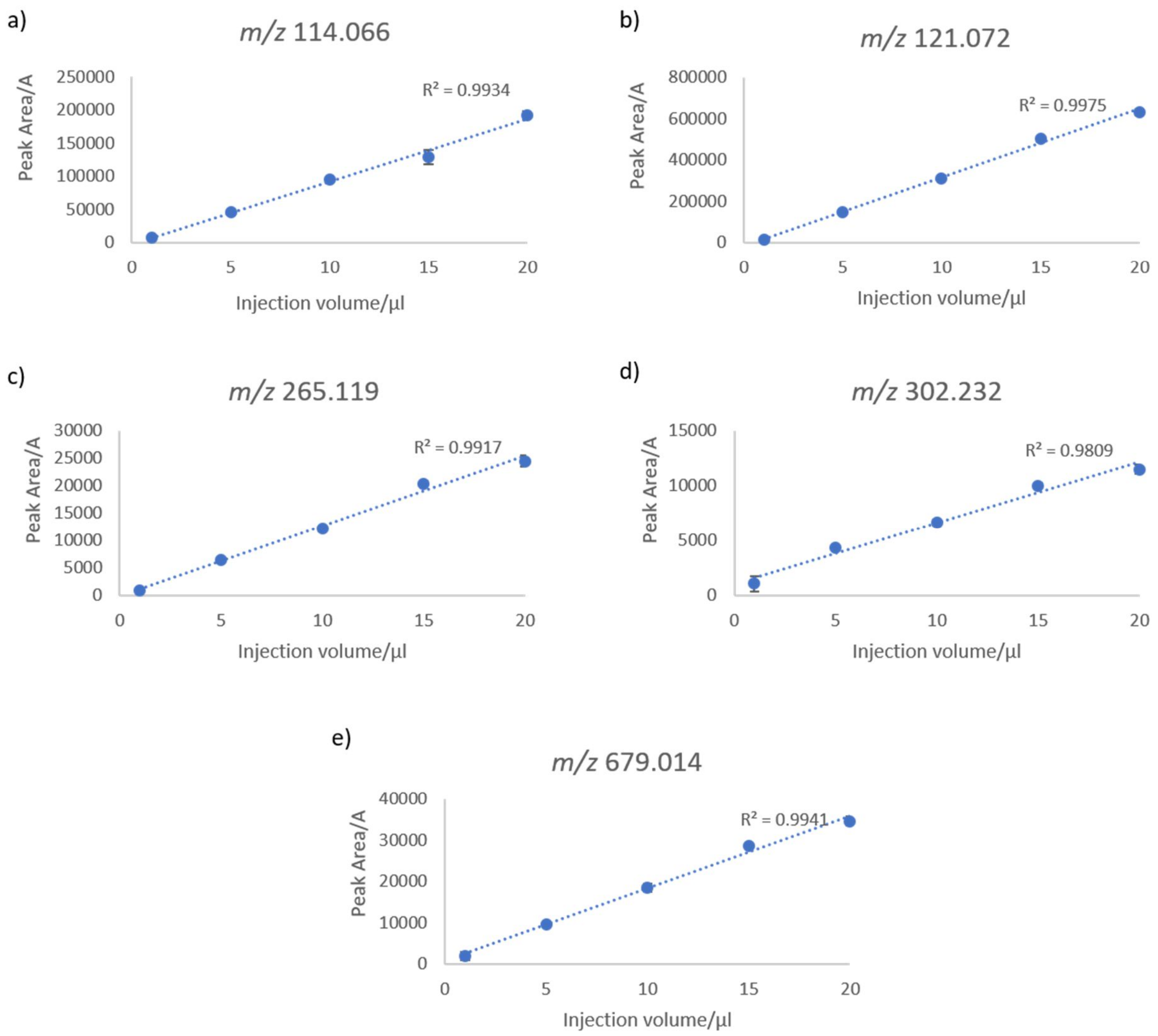

Fig. S1 Calibration graphs for selected ions for pooled urine sample. Injection volume range 1 – 20 µl in triplicates

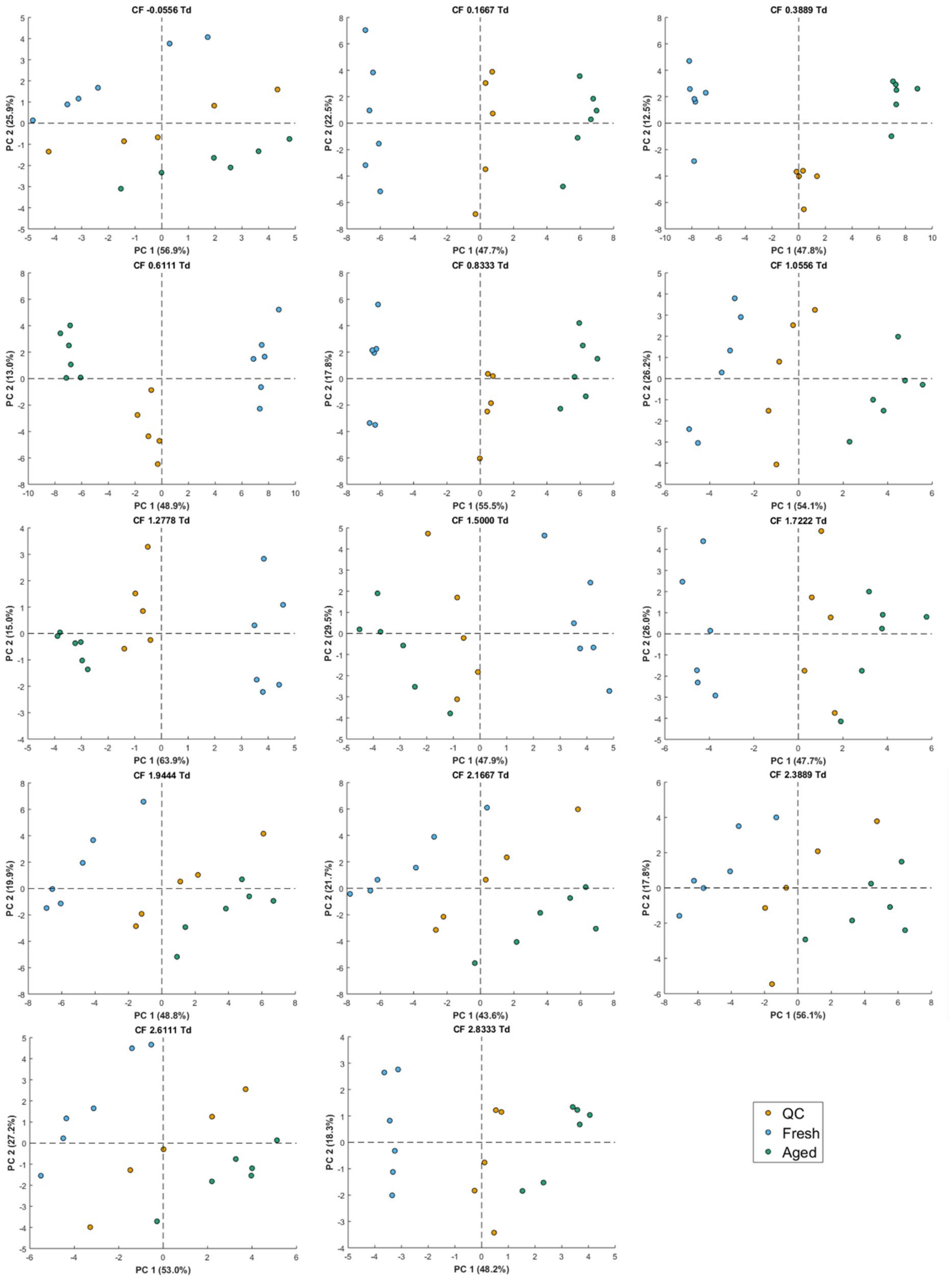

Fig. S2 PCA plots of urine samples containing fresh (blue), aged (green) and quality control samples (orange) at selected CF values

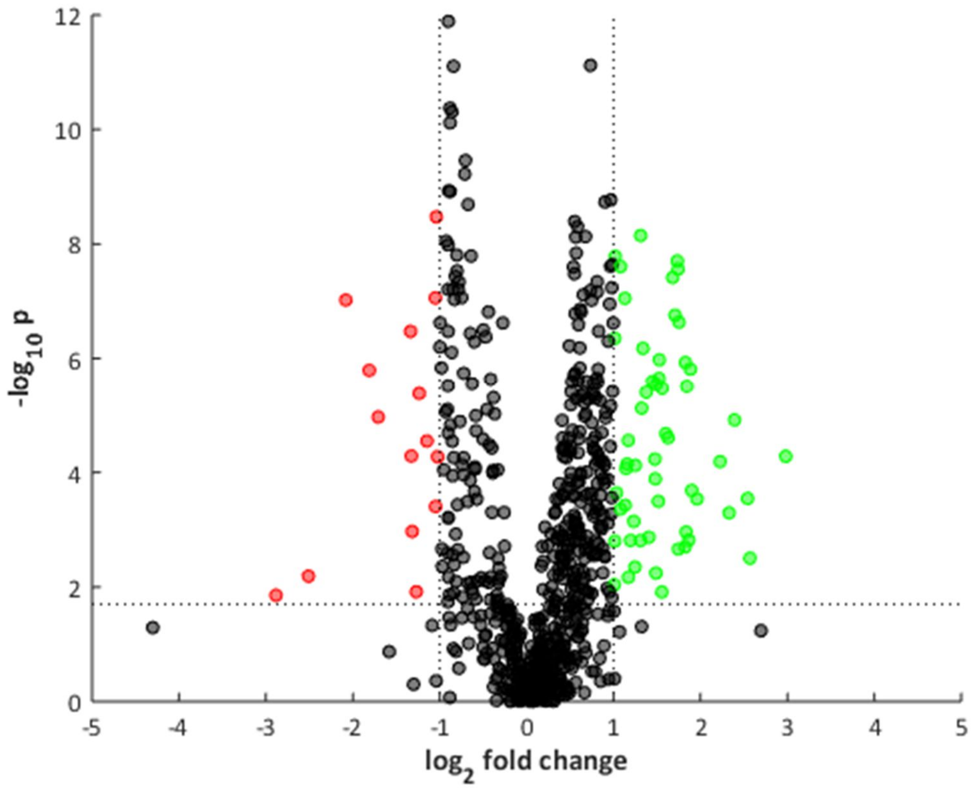

Fig. S3 Volcano plot of urine samples presenting ions with significant change in abundance between fresh and aged urine samples

Table S1 Ions with significant and at least two-fold change in abundance between fresh and aged urine samples

Mass measured	Retention time [min]	Median Fold change	p-value	0.011	CF (Td)
**104.1943**	9.02	3.04	2.08E-05	0.014	0.39
**108.081**	8.02	3.57	1.1E-03	0.018	-0.28
**110.055**	6.54	2.29	1.5E-03	0.038	-0.06
**114.0664**	3.41	2.80	5.7E-03	0.007	0.83
**116.1074**	5.16	2.05	2.2E-04	0.029	-0.28
**121.0718**	1.55	3.54	2.0E-03	0.005	0.83
**121.1644**	1.55	3.59	3.11-06	0.009	-0.28
**124.0723**	5.28	3.09	2.47E-05	0.007	-0.06
**131.1174**	8.32	5.25	1.21E-05	0.020	-0.06
**136.0395**	1.94	2.01	9.1E-03	0.036	0.17
**151.1444**	8.75	2.37	4.5E-03	0.008	0.83
**156.0777**	8.10	0.42	4.13E-06	0.009	0.39
**157.0611**	8.40	0.31	1.07E-05	0.004	0.39
**160.0967**	7.65	0.48	8.79E-08	0.021	0.17
**162.1119**	8.18	0.41	1.2E-02	0.017	1.94
**166.0723**	2.95	2.23	7.11E-05	0.001	1.50
**167.0571**	1.25	2.12	2.48 E-08	0.006	1.94
**181.0722**	0.99	2.59	3.93E-06	0.008	2.17
**185.1281**	2.41	2.94	3.70E-06	0.014	2.61
**191.1773**	8.67	2.38	7.47-05	0.019	0.83
**196.0962**	1.54	3.35	2.2E-03	0.011	-0.06
**203.1468**	7.94	0.49	5,.31E-05	0.002	-0.06
**207.1095**	2.41	3.69	1.54E-06	0.023	2.17
**212.0928**	5.97	0.17	6.4E-03	0.022	-0.06
**218.0213**	3.38	0.13	1.4E-02	0.015	0.17
**227.1264**	3.41	2.19	8.94E-08	0.002	1.06
**227.1789**	7.96	2.48	7.12E-09	0.018	-0.28
**229.1628**	7.44	0.40	5.15E-05	0.010	2.61
**229.2843**	7.41	0.45	2.82E-05	0.004	0.17
**231.2002**	8.61	2.02	4.47-07	0.014	0.61
**241.1547**	7.58	0.48	3.9E-04	0.003	0.39
**247.0956**	1.54	2.03	1.65E-08	0.003	0.17
**247.1461**	5.26	2.53	6.66E-07	0.007	-0.06
**248.1454**	7.84	0.40	3.37E-07	0.002	0.17
**257.1155**	2.41	2.88	1.06E-06	0.001	1.94
**262.1305**	8.45	0.49	3.36E-09	0.013	0.17
**269.1204**	1.43	2.35	7.15E-04	0.010	1.94
**283.1050**	2.19	2.19	3.74E-04	0.018	1.72
**286.1029**	1.99	0.40	1.07E-03	0.012	0.39
**286.2034**	5.62	2.21	8.50E-05	0.018	2.17

**287.1024**	4.04	2.02	1.58E-03	0.005	1.94
**290.1623**	8.50	0.24	9.53 E-08	0.008	0.17
**298.1145**	2.62	7.89	5.20E-05	0.019	1.94
**300.2018**	2.62	2.65	1.35E-03	0.004	2.39
**300.2176**	5.32	2.81	2.87E-06	0.011	2.39
**302.2028**	5.36	2.78	5.86E-05	0.015	2.39
**302.2331**	4.99	2.86	3.19E-04	0.010	2.17
**310.2031**	5.41	3.73	2.05E-04	0.001	1.94
**312.1335**	2.85	3.20	3.85E-08	0.013	2.17
**312.2202**	5.51	4.68	6.43E-05	0.028	2.39
**330.2343**	5.91	2.94	1.22E-02	0.011	0.61
**344.2331**	2.99	2.50	7.50E-06	0.018	2.83
**358.2600**	5.44	5.03	5.08E-04	0.009	2.17
**378.1819**	4.03	2.25	2.75E-05	0.022	2.39
**388.2567**	3.25	3.89	2.90E-04	0.002	2.83
**391.2341**	2.40	3.35	2.73E-08	0.009	2.39
**441.2404**	2.40	2.79	1.29E-04	0.004	1.94
**451.1645**	2.723	2.73	2.55E-06	0.006	1.94
**476.3110**	3.72	3.33	1.98E-08	0.007	2.83
**515.2209**	2.79	2.87	2.24E-06	0.023	1.50
**529.2289**	4.01	5.94	3.14E-03	0.003	2.17
**550.2043**	2.20	3.55	1.17E-06	0.002	1.94
**565.2099**	2.20	3.27	1.76E-07	0.016	1.72
**576.1310**	3.37	2.48	1.55E-03	0.021	1.94
**576.1669**	3.42	2.25	6.76E-03	0.013	1.72
**586.1524**	4.02	3.64	1.51E-03	0.016	1.72
**597.0168**	6.10	2.11	4.35E-04	0.009	2.17
**850.2716**	4.01	5.82	2.83E-04	0.006	1.72
**909.5473**	4.37	3.37	2.32E-07	0.022	1.50
**1194.1103**	1.11	0.28	1.61E-06	0.011	1.06
